# Surgical Outcome of Spontaneous Supra Tentorial Intracerebral Hemorrhage

**DOI:** 10.12669/pjms.334.12172

**Published:** 2017

**Authors:** Waqar Aziz Rehman, Muhammad Sohaib Anwar

**Affiliations:** 1 Dr. Waqar Aziz Rehman, FCPS General Surgery, FCPS neurosurgery Resident, Assistant Professor, Department of Neurosurgery, Sheikh Zayed Medical College/Hospital, Rahim Yaar Khan, Pakistan; 2 Dr. Muhammad Sohaib Anwar, FCPS Neurosurgery, Assistant Professor, Department of Neurosurgery, Sheikh Zayed Medical College/Hospital, Rahim Yaar Khan, Pakistan

**Keywords:** Hypertensive bleed, ICH, Intracerebral hemorrhage, Spontaneous bleed

## Abstract

**Objective:**

To assess the risks and benefits of surgical treatment (Open Craniotomy) of Intra-cerebral hematoma (ICH).

**Methods:**

Twenty seven patients of ICH who underwent surgical treatment at Neurosurgical department of Sheikh Zayed Hospital, Rahim Yar Khan, from 1^st^ January 2015 to 31^st^ December 2015 were included in this study. The primary outcome measured was death and improvement in GCS Status among survivor’s at three months.

**Results:**

Mean age of the patients was 58.4±10.7 and majority of patients (48.1%) were in the age range of 60-70 years. There were22.2% patients with ICH volume of >50 ml. Six (6) patients had 8 GCS with 50ml volume, who later died in ICU. Three of the patients who expired developed post-operative pneumothorax. These patients also acquired RTI resulting in deterioration of GCS. The rest of the expired patients showed deterioration in their GCS associated with oedma on brain CT scan. One patient died as a result of re-bleed. Twenty one (21) patients were discharged from hospital, two of these patients were lost in second follow up. Rest of the patients showed a gradual improvement in GCS touching 15/15 by 2^nd^ follow up visit.

**Conclusion:**

Surgical prognosis of ICH depends on the patients GCS received and size of hemorrhage at the time of presentation. Urgent surgical evacuation in patients with rapid deterioration carries good outcome, hence should be considered.

## INTRODUCTION

Intracerebral hemorrhage (ICH) accounts for 10 to 20% of strokes in Caucasians and Europeans, with statistics reaching 20-30% in Asian and black population.^1^,^2^ It is the third leading cause of death and main cause of long term disability.^3^

Hypertensive arteriosclerosis is responsible for 80% of primary hemorrhages followed by other causes like vascular malformations and Amyloid Angiopathy. Poorly controlled hypertension is often found in most ICH patients and at times it may be difficult to identify the underlying etiology.^4^

The pathophysiology of primary ICH is, chronic high pressure leading to hemorrhage from small perforating arteries, typically at bifurcations, where pressure gradient is transmitted from larger vessels to smaller susceptible vessels. This explains why ICH occurs frequently in deep grey matter structures most commonly basal ganglia followed by Thalamus, Cerebellum, and Pons and rarely lobar. Computed tomography (CT) Brain is a rapid sensitive means to diagnose ICH in initial phases.^5^

A lot of efforts have been put forward in search for the ideal surgical procedure of ICH but still a wide variation in the management of PICH is seen among different Neurosurgeons of the world and even in same centers.^6^

The ideal goal of surgical treatment of ICH is to remove as much blood clot as possible and as quickly as possible with the least amount of brain trauma from surgery itself. These surgical techniques includes CT guided stereotaxic aspiration, simple aspiration of ICH through a burr hole and open craniotomy each having their own merits and demerits.^7^ The aim of this study was to assess the risks and benefits of surgical treatment (Open Craniotomy) of Intra-cerebral hematoma (ICH) in our set up and consider the implications of theresults on current clinical practice and future research endeavors.

## METHODS

This descriptive study was conducted in the Neurosurgical department of Sheikh Zayed Hospital, Rahim Yar Khan, from 1^st^ January 2015 to 31^st^ December 2015. During this time span 27 patients were included. Most of the patients were admitted from the Medical, Neurology and Emergency departments. The patients, who were received by the respective departments, underwent their own initial assessment, clinical examination, initial management and CT scan of the brain. On confirmation of ICH, the departments called us for evaluation of surgical management of the hemorrhage.

### Inclusion and Exclusion criteria

The patients with Spontaneous, hypertensive, Supratentorial bleeds, having GCS 8-13, with Vol. 20ml and above and having age 18-70 yearswere included in the study. While Patients of ICH having 14-15 GCS were not included in the study. Patients diagnosed as AVM/aneurysms, associated Intra ventricular bleed, tumor bleed, post-operative bleeds, Patients taking anticoagulants, and those received with initial Glasgow Coma Scale(GCS) below 7 were excluded from the study.

In patients with unusual site of bleed further investigation like CT Scan Angiography. I/V contrast (Brain) was done to rule out arterio-venous malformations (AVM), aneurysm, tumor bleed etc.

The patient who met the incluisoncriteria underwent surgery and were kept in ICU postoperatively, where serial CT Scans were done, when required. The patients who were discharged were followed for three months, with three visits in OPD at 15 days, one month, and three months. The primary outcomemeasured was death and improvement in GCS Status among survivor’s at three months.

The data was maintained on a pre-designed Performa mentioning variables like age, sex, diagnosis, previous medical history, site of the lesion, GCS (pre, post, at discharge, at each follow up) and mortality, etc. SPSS v23 software was used for data compilation and calculation of mean and standard deviations and percentage.

## RESULTS

This study was carried out on 27 patients. Mean age of the patients was 58.4±10.7 and majority of patients (48.1%) were in the age range of 60-70 years. There were 70.4% males in this study and 52.8% patients were having volume of Intracerebral hemorrhage (ICH) 20-40 ml, 25.9% with hemorrhage volume 40-50 ml and 22.2% with hemorrhage volume >50 ml. location of Intra-cerebral hemorrhage was basal ganglion in 85.2% patients and lobar areas in 14.8% patients (Table-I).

**Table-I:** Pre-procedural characteristics of ICH Patients.

*Variable*	*Value*
Mean Age (Years)	58.4±10.7

***Age Distribution (%)***	

60-70 Years	13 (48.1)
50-59 Years	12 (44.4)
40-49 Years	2 (7.4)
18-39 Years	Nil
Male Gender (%)	19 (70.4)
Female Gender (%)	8 (29.6)

***Volume of Intracerebral Hemorrhage (%)***	

20-40 ml	14 (52.8)
40-50ml	7 (25.9)
50+ ml	6 (22.2)

***Location of Intracerebral Hemorrhage (%)***	

Basal Ganglion region	23 (85.2)
Lobar Areas	4 (14.8)

Six patients having ICH volume greater than 50ml were unable to survive and post operatively scummed due to rebleed and other complications. Three of the patients who expired developed pnemothoracic, 3-7 day post operatively for which a chest tube was placed. These patients also acquired RTI resulting in deterioration of GCS. The rest of the expired patients showed deterioration in their GCS associated with oedma on their CT scan brain, with one patientwho died as a result of re-bleed. ICH volumes between 40-50ml showed promising results as far as improvement of GCS is concerned.

The patients on admission were grouped in three, according to the GCS received. Six patients of ours had 8 GCS with 50ml+volume, who later died in ICU. 55% of the patients had GCS 9-10, 13 of them gradually improved and normalized by the 3^rd^ follow up. Similarly six patients having GCS 11-13 regained 15 GCS by the second follow up, respectively (Table-II).

**Table-II:** Presenting GCS of Operated Patients.

*GCS*	*Number of Patients*			
	< 8 GCS	9-10 GCS	11-13 GCS	14-15 GCS
Pre-Op	6 (22.2)	15 (55.6)	6 (22.2)	-----
Post-Op	6 (22.2)	8 (29.6)	13 (48.2)	-----
At the time of Discharge	-----	------	21 (77.7)	-----
1^st^ Follow Up	-----	------	5 (18.5)	16 (59.2)
2^nd^ Follow Up	-----	------	-----	19 (70.37)
3^rd^ Follow UP	-----	------	-----	19 (70.37)

In-hospital mortality rate was 6 (22.2%) and remaining 21 (77.8%) were discharged from the hospital. Out of these 21 patients, two patients were lost in our second follow up. The rest of them showed a gradual improvement in GCS touching 15/15 by 2^nd^ follow up visit.

## DISCUSSION

The outcome of the 27 series of surgically treated patients with spontaneous Intracerebral hemorrhage is potentially confounded because the control groups with which they were compared were not similar. The factors (besides treatment) that may have influenced patient outcomeincluded, patient’s age, level of consciousness, severity of limb paresis, continence status, the site, size, volume, mass effect of the haemorrhageand the presence or absence of blood into the ventricles or subarachnoid space.

Inagawa et al. had done a similar study and according to his study the most common site of ICH was the putamen (34%), followed by the thalamus (33%), lobar areas (15%). They concluded that short-term and long-term outcomes after ICH were directly related to the site of hemorrhage and the severity of bleeding, which was assessed by the hematoma volume and Glasgow Coma Scale score. In their study, overall mortality rate was 54%.^8^

Hossian et al. concluded that GCS <8 and volume of hematoma more than 60 mm is associated with poor outcomes after surgery. In their study death rate was 31.0%.^9^ In our study, death occurred in 22.2% patients, and all of these patients were with pre-operative GCS score 8 and were with hematoma volume >50 ml.

In the study of Hossain et al. 71% patients had location of ICH in basal ganglion region and only 29% in lobar areas.^9^ In our study, ICH of basal ganglion region was diagnosed in 85.2% patients and that of lobar region in 14.8% patients. Broderick et al. also concluded that pre-operative hemorrhage volume and GCS score is a stronger predictor of operative mortality in patients with ICH.^10^

The STICH trial published in 2005, excluded patients having GCS score >5 and concluded that patients having GCS score 5-8 are associated with 91.0% risk of poor outcomes.^11^ Yelmez et al. conducted a study on 25 comatose patients having GCS ≤8. The success rate of craniotomyin their study was 44% and the authors concluded that early surgical treatment should be 1^st^ line treatment of choice in selected patients with poor GCS score. This study also concluded that hematoma volume >60 ml is associated with worst surgical outcomes and surgical outcomes are better in patients with poor GSC score but with smaller hematoma volume.^12^ In our study, all patients having GCS score 8 died and all of these patients were having hematoma volume >50 ml. Some other studies have also shown similar results.^13^,^14^ Our study results are comparable to these studies.

## CONCLUSION

Surgical prognosis of ICH depends on the patients GCS received and size of hemorrhage at the time of presentation. Urgent surgical evacuation in patients with rapid deterioration carries good outcome, hence should be considered.

## References

[1] Tak Fai Cheung R (2007). Update on medical and surgical management of intracerebral hemorrhage. RevRecent Clin Trials.

[2] Rasool A, Rahman A, Choudhury S, Singh R (2004). Blood pressure in acute intracerebral haemorrhage. JHum Hypertens.

[3] Mitchell P, Gregson BA, Vindlacheruvu RR, Mendelow AD (2007). Surgical options in ICH including decompressive craniectomy. J NeurolSci.

[4] Kumar NSS, Neeraja V, Raju CG, Padala RK, Kumar TA (2015). Multiple spontaneous hypertensive intracerebral hemorrhages. J Stroke Cerebrovasc Dis.

[5] Walshe TM, Hier DB, Davis KR (1977). The diagnosis of hypertensive intracerebral hemorrhage: The contribution of computed tomography. Comput Tomogr.

[6] Lejeune J, Thines L (2003). Neurosurgical management of spontaneous cerebral hemorrhage. J Neuroradiol.

[7] Javed G, Tahir MZ, Enam SA (2008). Role of neurosurgery in the management of stroke. J Pak Med Assoc.

[8] Inagawa T, Ohbayashi N, Takechi A, Shibukawa M, Yahara K (2003). Primary intracerebral hemorrhage in Izumo City, Japan: incidence rates and outcome in relation to the site of hemorrhage. Neurosurgery.

[9] Hossain M, Ahmed S, Ansary S, Islam S (2010). Surgical Outcome Of Spontaneous Intracerebral Haematoma Through Keyhole Craniectomy. Faridpur Med Coll J.

[10] Broderick JP, Brott TG, Duldner JE, Tomsick T, Huster G (1993). Volume of intracerebral hemorrhage. A powerful and easy-to-use predictor of 30-day mortality. Stroke.

[11] Mendelow AD, Gregson BA, Fernandes HM, Murray GD, Teasdale GM, Hope DT (2005). Early surgery versus initial conservative treatment in patients with spontaneous supratentorial intracerebral haematomas in the International Surgical Trial in Intracerebral Haemorrhage (STICH): a randomised trial. Lancet.

[12] Yilmaz C, Kabatas S, Gulsen S, Cansever T, Gurkanlar D, Caner H (2010). Spontaneous supratentorial intracerebral hemorrhage: Does surgery benefit comatose patients?. Ann Indian Acad Neurol.

[13] Hemphill JC, Bonovich DC, Besmertis L, Manley GT, Johnston SC (2001). The ICH score a simple, reliable grading scale for intracerebral hemorrhage. Stroke.

[14] Pai SB, Varma R, Parthiban J, Krishna K, Varma R, Srinivasa R (2007). Keyhole craniectomy in the surgical management of spontaneous intracerebral hematoma. Neurol Asia.

